# Management of a traumatic superior mesenteric artery injury using superselective angioembolization

**DOI:** 10.1016/j.jvscit.2025.101726

**Published:** 2025-01-15

**Authors:** Martha Ennis, Jurgienne Umali, David Pace

**Affiliations:** Discipline of Surgery, Faculty of Medicine, Memorial University of Newfoundland, St. John's, Newfoundland and Labrador, Canada

**Keywords:** Blunt trauma, Angioembolization, Superior mesenteric artery injury, SMA injury

## Abstract

We report the successful use of angioembolization to treat mesenteric bleeding in a patient who sustained blunt abdominal trauma. Angiography revealed extravasation from a distal branch of the right colic or ileocolic artery. Interventional radiology superselectively embolized a distal arterial branch supplying the ileum. Total hospital stay was 4 days. Laparotomy is the standard treatment for active mesenteric bleeding. This case highlights the usefulness of superselective embolization to mitigate the need for surgical intervention in a patient with active mesenteric bleeding. Treatment outcomes depend on close clinical monitoring for intestinal ischemia and patient counselling on the potential for surgical intervention.

Mesenteric arterial injuries are rare and occur in 1% to 5% of trauma patients.^1^ In Canada, 28 cases of traumatic superior mesenteric artery (SMA) injuries were reported across 8 level 1 trauma centers between 2011 and 2015, accounting for 2% of reported traumatic vascular injuries.^2^ Despite their rarity, high mortality rates (≤43%) from SMA injuries have been reported owing to technical difficulties in obtaining vascular control and the vessel's capacity for robust blood flow leading to early exsanguination, hemorrhagic shock, and multiorgan failure.^3^ Although surgery is the standard treatment for active mesenteric bleeding,^2^^,^^3^ there are a number of cases managed with arterial embolization that have been reported in the literature.^4^, ^5^, ^6^, ^7^, ^8^, ^9^

We report the successful use of angioembolization in a 63-year-old man with mesenteric bleeding secondary to blunt abdominal trauma. The patient provided consent for publication of their case details and images.

## Case report

A 63-year-old man presented to the emergency department with a 1-day history of diffuse abdominal pain and persistent rectal bleeding after being kicked by a Newfoundland pony in the abdomen. He went to the emergency room the following day because of persistent and worsening symptoms. He was taking rivaroxaban for atrial fibrillation, and the last dose was taken the day before his visit to the emergency room.

The patient also had a past medical history of hyperlipidemia and benign prostatic hyperplasia. He had no surgical history. His medications included rivaroxaban, metoprolol, rosuvastatin, and tamsulosin. He was a nonsmoker with no known allergies.

Initial vital signs in the emergency department revealed a tachycardia of 115 to 130 bpm with an irregular rhythm. Blood pressure was stable in the 120s mm Hg systolic. Temperature was 36.9 °C, respiratory rate was 16, and oxygen saturation was 100% on 3 L via nasal prongs. On physical examination, the patient seemed to be unwell and was pale and diaphoretic. His abdomen was distended with rebound tenderness. One gram of tranexamic acid was administered intravenously. A focused assessment of sonography in trauma examination found free intraperitoneal fluid.

**Fig 1:**
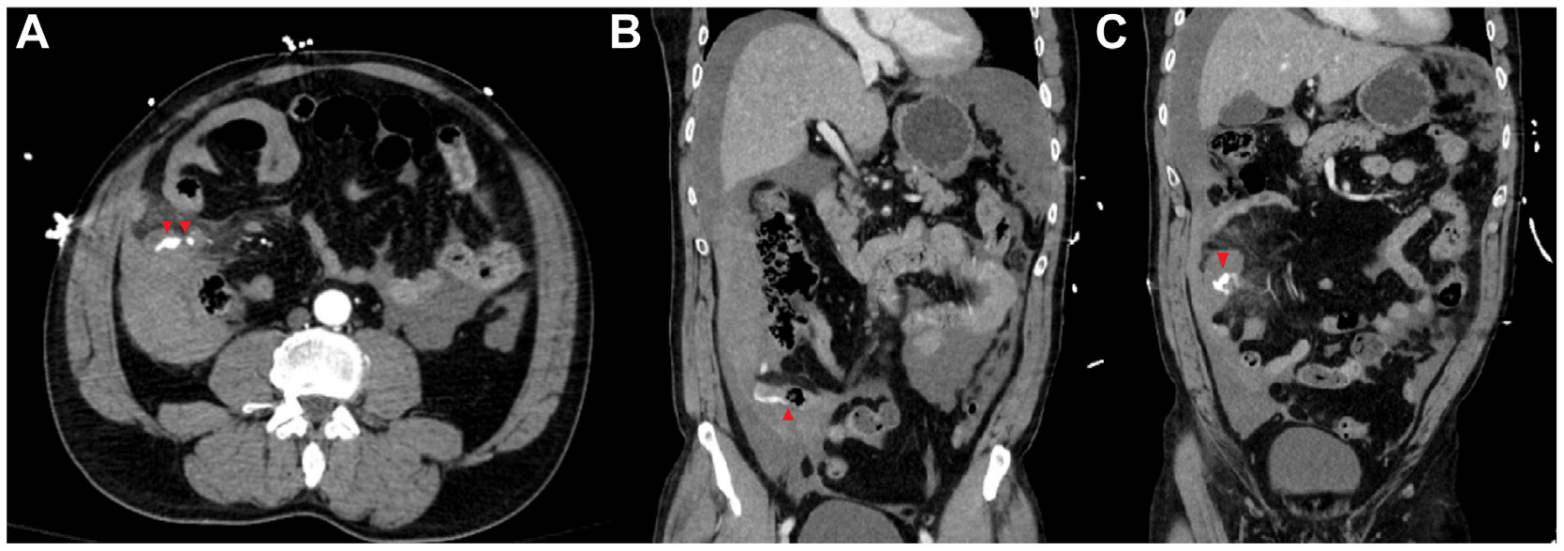
Computed tomography angiography of the abdomen and pelvis demonstrating active arterial contrast extravasation favored to be from a distal branch of either the right colic or ileocolic artery off the superior mesenteric artery (SMA). There is a small ovoid hematoma at the site of extravasation measuring up to 3.5 cm **(A)**. Adjacent to the described arterial bleed, there is pooling of arterial contrast on the delayed phase within a short segment of ileum **(B)**, which seems to be separate from the arterial bleed in the distal branch of the SMA immediately above it **(C)**.

Laboratory investigations revealed a leukocyte count of 14.5 × 10^9^ cells/L, a hemoglobin of 107 g/L, and a platelet count of 198 × 10^9^ cells/L. Lactate was 9.43 mmol/L. Urea was 8.6 mmol/L, and creatinine was 114 μmol/L. Electrolytes were within normal limits, and the international normalized ratio was 1.47. 1computed tomography angiography revealed active contrast extravasation in the right lower quadrant, favored to be a distal branch of either the right colic or the ileocolic artery (Fig 1, *A*). There was marked mesenteric stranding around the area of active extravasation and moderate volume hemoperitoneum. Adjacent to the described arterial bleed, there was pooling of arterial contrast on the delayed phase within a short segment of ileum (Fig 1, *B*), seeming to be separate from the arterial bleed in the distal branch of SMA immediately above it (Fig 1, *C*). Several bowel loops in the region of mesenteric stranding/active extravasation were collapsed and appeared slightly ill-defined with questioned mildly reduced enhancement. There was no evidence of free air or perforation.

General surgery was consulted, and the team decided to refer the patient to interventional radiology for possible angioembolization. The potential need for surgery was also discussed with the patient.

**Fig 2:**
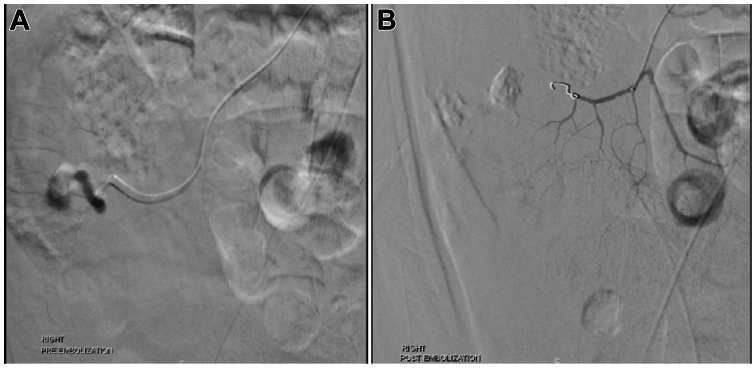
Superior mesenteric arteriogram before and after embolization of the actively bleeding SMA injury. Active extravasation was identified arising from the branch supplying the ileum **(A)**. A 5-minute delay repeat angiogram was performed after selective catheterization and coiling of this distal branch, which showed no further extravasation **(B)**.

After obtaining informed consent, vascular access was obtained via the right common femoral artery, and a 5F vascular sheath was inserted. A 5F pigtail catheter was advanced into the upper abdominal aorta, and an aortogram was performed, outlining the intra-abdominal arterial anatomy. Minimal extravasation was noted in the right lower quadrant at that time. The pigtail catheter was then replaced with a 5F C3 catheter, and the SMA was selected and catheterized. SMA arteriogram was then performed, and active extravasation from a branch supplying the ileum was identified (Fig 2, *A*). Superselective catheterization and embolization were then performed. Through the C3 catheter, a 2.7F Progreat catheter was inserted. The distal branch supplying the hemorrhage was selectively catheterized. A single 3 mm × 2.5 mm fibered coil was deployed. After a 5-minute delay, a repeat angiogram was performed, and this showed no further extravasation (Fig 2, *B*). Catheters were removed, and hemostasis was obtained using manual pressure for 10 minutes.

After this procedure, 1000 units of prothrombin complex concentrate (PCC), and 1 U of packed red blood cells were administered intravenously. The international normalized ratio after administration of PCC was 1.29. Hemoglobin repeated 8 hours after the procedure was 110 g/L. The patient was admitted and monitored for signs of intestinal ischemia. His hemodynamic status normalized, his abdominal pain improved, and his rectal bleeding stopped. Repeat hemoglobin was 86 g/L on postprocedure day 1; lactate was 2.81 mmol/L. Hemoglobin was drawn later that day, and came back at 82 g/L. On postprocedure day 2, hemoglobin decreased to 75 g/L, and another unit of packed red blood cells was administered. His hemoglobin increased appropriately after the transfusion to 92 g/L and remained stable on repeat. An abdominal radiograph performed to investigate abdominal distension and lack of stooling was in keeping with ileus and no free air. He was managed expectantly and tolerated advancement to a regular diet over the course of 2 days once bowel function was restored. The total length of stay was 4 days.

At the 2-week outpatient follow-up, the patient was clinically well and reported only mild abdominal pain with activity. He had normal bowel function. He sold his pony.

## Discussion

SMA injuries are rare but carry an estimated overall mortality risk of 43%, with little improvement in survival across the literature.^3^ SMA injuries are classified as per the Fullen classification, which is based on anatomical zone and ischemia grade.^10^ A more proximal injury comes with an increased number of bowel segments at risk of ischemia. Zone 1 trauma involves the trunk proximal to the inferior pancreaticoduodenal artery, the SMA's first major branch.^11^ Zone 2 trauma involves the region between the inferior pancreaticoduodenal and middle colic artery, and zone 3 involves the SMA trunk distal to the middle colic artery.^11^

Our patient had an injury in Fullen zone 4, which involves segmental branches, jejunal, ileal, or colic segments of the SMA.^10^ They typically cause minimal to no ischemia, although extensive ischemia may occur if multiple segments are injured.^10^ Injuries in Fullen's anatomical zone IV have been found to carry a risk of approximately 23% mortality and are either repaired primarily or ligated.^11^ The patient's rectal bleeding and elevated lactate upon presentation could have been signs of bowel ischemia, although it did cease after endovascular intervention. Close monitoring is crucial after embolization given the risk of ischemia, with serial physical examinations, vital signs monitoring, and blood tests.^6^ The patient's abdominal pain improved, and lactate levels decreased when repeated after the procedure. Repeat computed tomography scan with intravenous contrast 24 to 48 hours after the procedure, regardless of clinical status has been performed in the literature^6^^,^^7^; however, we did not perform it, although it could be considered in the future.

Although the gold standard treatment in mesenteric injury with active bleeding is operative management, there have been cases in the literature highlighting the use of transcatheter arterial embolization (TAE), with the goal of hemostasis and limiting the formation of hematoma that could impair intestinal vascularization.^6^ It has been used in both unstable and stable patients in the literature,^7^^,^^8^ and although the sample sizes remain small, technical success mitigates the need for surgical intervention. A literature review of nine articles on blunt traumatic mesenteric bleeding treated with TAE between 1994 and 2019 demonstrated that a common complication is intestinal necrosis which required surgical intervention.^5^, ^6^, ^7^ In total, analysis of 25 cases done by Bertelli et al^6^ showed a TAE technical success rate of 96%, with surgical intervention being performed in 28% of cases, with a higher failure rate in elderly patients, as well as patients with multiple lesions, mesenteric bleeding in the mesocolon, and the use of coils. Of note, a multi-institutional study looking at SMA injuries showed that Fullen's zones 1 and 2 and Fullen's ischemic grade of 1 are highly predictive of mortality, with Fullen zone 1 injuries having a mortality of more than 70%, and zone 2 of 44.1%, although the patients involved had concomitant vascular and nonvascular injuries.^11^ This finding indicates that proximal SMA injuries, particularly Fullen 1 lesions, should be repaired operatively owing to the mortality associated with midgut ischemia. The rarity of SMA injuries and cases reported, along with the change in technologies used and resource availability in the past 25 years, results in the lack of uniformity of management and monitoring after TAE.^6^ Thus, we hope our case report helps to address the paucity of the literature on the nonoperative management of SMA injuries.

Of note, this patient underwent a mesenteric arterial intervention and is considered a high procedure-associated bleeding risk,^12^ in addition to the risks that come with being on a direct oral anticoagulant. For a patient taking rivaroxaban undergoing an emergent procedure with a high bleeding risk, reversal with andexanet alfa is recommended,^12^ although this medication was not available at our institution at the time. The patient did receive PCC, which is recommended in guidelines for patients receiving direct factor XA inhibitors presenting with severe or life-threatening bleeding.^13^ Andexanet alfa was approved by Health Canada for the treatment of patients with life-threatening or uncontrolled bleeding who require reversal of rivaroxaban or apixaban in mid August 2023,^14^ which will change the clinical practice with regards to direct oral anticoagulant management in bleeding patients. Postprocedure, hemostasis was obtained after the removal of femoral access catheters by applying manual pressure for 10 minutes. Percutaneous closure devices are used routinely for accesses requiring material above 6F, patients under anticoagulant treatment or with impaired blood coagulation, and could have provided a shorter time to hemostasis, mobilization and greater patient comfort, and have been shown to reduce the number of hematomas larger than 5 cm compared with manual compression.^15^

As demonstrated in this case, embolization is reasonable in the setting of blunt abdominal trauma and active mesenteric bleeding with no evidence of bowel perforation. Depending on the location and extent of mesenteric injury, resource availability and how quickly interventional radiology can be mobilized, embolization could potentially be considered as first-line treatment for active mesenteric bleeding. Close clinical monitoring is required, and one must counsel the patient on potential need for emergent surgical intervention in the event of failure.

## Conclusions

This case highlights the use of superselective embolization to mitigate the need for surgical intervention in appropriately selected patients with mesenteric bleeding following blunt abdominal trauma. Treatment success depends on close clinical monitoring after the procedure, and patient counselling on potential need for operative intervention in the case of rebleeding or ischemia.

## Funding

None.

## Disclosures

None.
